# Supplementary material for: Deriving the Dietary Approaches to Stop Hypertension (DASH) Score in Women from Seven Pregnancy Cohorts from the European ALPHABET Consortium
Source: Nutrients. 2019 Nov 8;11(11):2706. doi: 10.3390/nu11112706 (PMC6893477; doi:10.3390/nu11112706)
Supplement: Supplementary file 1 [file nutrients-11-02706-s001.pdf]

nutrients

MDPI

Table S1. FFQ frequency scales of the ALPHABET consortium.

Cohort	ALSPAC	EDEN	Generation R	Lifeways	REPRO_PL	ROLO	SWS
Total number of categories	5	7	9	9	6	9	≥8
Specific categories	Never or rarely	Never	Never or less than once a month	Never or less than once a month	Never	Never or less than once a month	Never
		Less than once a month			Less than once a month		Once every 2–3 months
	Once in 2 weeks	1–3 times a month	1–3 times a month	1–3 times a month	1–3 times a month	1–3 times a month	Once a month
							Once a fortnight
	1–3 times a week	Once a week	Once a week	Once a week	1–3 times a week	Once a week	1–2 times a week
		2–5 times a week	2–4 times a week	2–4 times a week		2–4 times a week	
	4–7 times a week				4–6 times a week		3–6 times a week
		Once a day or nearly	5–6 times a week	5–6 times a week		5–6 times a week	
			Once a day	Once a day	Everyday	Once a day	Once a day
	More than once a day	Several times a day	2–3 times a day	2-– times a day	Not applicable	2–3 times a day	More than once a day (open)
			4–5 times a day	4–5 times a day		4–5 times a day	
			More than 6 times a day	6 times a day or more		6 times a day or more	

Table S2. Summary of included and excluded foods for each food components composing the DASH index.

Component	Summary of included foods	Summary of excluded foods
**Positive components**		
Dietary components for which greater intakes receive higher scores		
Total grains	• Refined and unrefined grains and grain products *(bread, pasta, rice, etc.)*	• Salty and sweet grain products *(bun, cheese crackers, etc.)*<br>• Foods with grains as base only *(ravioli, pizza, pudding, etc.)*
Vegetables (excluding potatoes and condiments)	• Fresh, frozen, canned vegetables, vegetables juices	• *Potatoes*<br>• Small portion condiments *(garlic, parsley, etc.)*
Fruits	• Fresh, frozen, dried and canned fruits, fruit juices	• Fruit drinks not considered as fruit juices
Non-full-fat dairy products	• All milks and milk products undeclared as full-fat or full-cream<br>• All cheeses declared as fat-free or low-fat	• All milks and milk products declared as full-fat or full-cream<br>• All cheeses undeclared as fat-free or low-fat<br>• Milk substitutes
Nuts, seeds and legumes	• *Nuts and peanut butter, dried beans, peas, tofu, etc.*	
**Negative components**		
Dietary components for which lower intakes receive higher scores (Reverse scoring)		
Red and processed meats		• Not full of meat ready meals *(meat pies, meat soup, etc.)*
Sugar-sweetened beverages, sweets and added sugars	• Soft drinks, added sugar to drinks and meals *(coffee/tea, yoghurt)*, sweets, etc.	• Artificial sweeteners *(diet coke, stevia, etc.)*<br>• Sweets with fats and savoury snacks *(cocoa spreads, mars, etc.)*
Sodium	• All sodium consumption estimated	

Table S3. Spearman's correlation coefficients (rho (95% CI)) between DASH scores from frequencies and amounts, by cohort and period in ALPHABET.

	Pre-pregnancy period	Early pregnancy period	Late pregnancy period
**EDEN**	0.89 (0.88, 0.90)	-	0.89 (0.88, 0.90)
**Lifeways**	-	0.92 (0.91, 0.93)	-
**ROLO**	-	0.88 (0.86, 0.89)	-

All $p < 0.0001$. DASH: Dietary Approaches to Stop Hypertension. -: data not available. ALSPAC, Generation R, REPRO_PL and SWS do not have DASH scores from both frequencies and amounts.
